# Clinical consequences of untreated dental caries in German 5- and 8-year-olds

**DOI:** 10.1186/s12903-015-0121-8

**Published:** 2015-11-04

**Authors:** Katrin Grund, Inka Goddon, Ina M. Schüler, Thomas Lehmann, Roswitha Heinrich-Weltzien

**Affiliations:** 1 Department of Preventive and Paediatric Dentistry, Jena University Hospital, Bachstr. 18, D-07743 Jena, Germany; 2 Department of Social Services and Health, Health Services for Children and Adolescents Schwelm, Hauptstr. 92, D-58332 Schwelm, Germany; 3 Department of Medical Statistics and Epidemiology, Jena University Hospital, Bachstr. 18, D-07743 Jena, Germany

**Keywords:** Children, Odontogenic infections, Primary dentition, pufa index, Untreated dental caries

## Abstract

**Background:**

About half of all carious lesions in primary teeth of German 6- to 7-year-old children remain untreated, but no data regarding the clinical consequences of untreated dental caries are available. Therefore, this cross-sectional observational study aimed to assess the prevalence and experience of caries and odontogenic infections in the primary dentition of 5- and 8-year-old German children.

**Methods:**

Dental examinations were performed in 5-year-old pre-school children (*n* = 496) and in 8-year-old primary school children (*n* = 608) living in the Westphalian Ennepe-Ruhr district. Schools and preschools were selected by sociodemographic criteria including size, area, ownership, socio-economic status. Caries was recorded according to WHO criteria (1997). The Lorenz curves were used to display the polarisation of dental caries. Caries pattern in 5-year-olds was categorized by Wyne’s (1997) definition of early childhood caries (ECC). Odontogenic infections as clinical consequence of untreated dental caries were assessed by the pufa index. The ‘untreated caries-pufa ratio’ was calculated, and the Spearman’s rank correlation coefficient (ρ) was used for evaluating the correlation between dmft and pufa scores. Categorical data were compared between groups using the chi-square test and continuous data were analysed by t-test.

**Results:**

Caries prevalence and experience in the primary dentition was 26.2 %/0.9 ± 2.0 dmft in 5-year-olds and 48.8 %/2.1 ± 2.8 dmft in 8-year-olds. ECC type I (22 %) was the prevalent caries pattern in 5-year-olds. About 30 % of the tooth decay was treated (5y: 29.7 %/8y: 39.3 %). The Lorenz curves showed a strong caries polarisation on 20 % of the children. Pufa prevalence and experience was 4.4 %/0.1 ± 0.5 pufa in 5-year-olds and 16.6 %/0.3 ± 0.9 pufa in 8-year-olds. In 5-year-olds 14.2 % and in 8-year-olds 34.2 % of the d-component had progressed mainly to the pulp. A significant correlation between dmft and pufa scores exists in both age groups (5y: ρ = 0.399; 8y: ρ = 0.499). First deciduous molars were most frequently affected by odontogenic infections, presenting virtually all pufa scores (>95 %).

**Conclusions:**

Prevalence and experience of odontogenic infections and the untreated caries-pufa ratio were increasing from the younger to the elder children. Dmft and pufa scores in primary teeth predict a higher caries risk in permanent teeth. The pufa index highlights relevant information for decision makers to develop effective oral health care programs for children at high risk for caries.

## Background

Dental caries continues to be one of the most widespread diseases in the world [1]. In particular, children are predisposed to the development of carious lesions, and their treatment is not just a problem in low- and middle-income countries [2, 3]. Even in economically developed countries such as the United Kingdom, France, Germany, and the United States, the treatment of decayed primary teeth remains an on-going public health challenge [4–7].

Severely decayed teeth have an important impact on children’s general health, nutrition, growth and body weight [8–10] by causing discomfort, pain, sleeping problems, learning disorders and absence from school [11–13]. Furthermore, odontogenic infections as a consequence of untreated dental caries are the most frequent reason for the hospitalisation of young children [6, 14]. Therefore, oral health fundamentally influences children’s general health and quality of life [15–17].

The early onset of dental caries is of exceptional concern since it represents an indicator for missed opportunities for preventive care and endangers the general health of a child. Wyne classified dental caries in children aged younger than 6 years as early childhood caries (ECC) by three types of severity [18]. Type I has been defined as the existence of isolated carious lesion(s) on molars and/or incisors, type II as labiolingual carious lesions on maxillary incisors and type III as carious lesions on almost all teeth, including lower incisors [18]. The American Academy of Pediatric Dentistry (AAPD) defines ECC as the presence of one or more decayed (non-cavitated or cavitated lesions), missing (due to caries), or filled tooth surfaces in any primary tooth in a child under the age of six [19].

Most epidemiological studies performed in children have used the dmft index, which provides information on the caries experience and restorative and surgical treatment, but fails to contribute data on the consequences of untreated caries. The diagnosis ‘*teeth indicated for extraction*’ is a WHO criterion for treatment needs [20], but does not give detailed information about the severity of advanced caries lesions and is rarely used in the literature.

In 2010, Monse et al. [21] introduced a new clinical index characterising the consequences of untreated dental caries in primary and permanent teeth: the pufa/PUFA index. It is calculated as sum of teeth with four diagnoses concerning different kinds of odontogenic infections ([p] pulpal involvement, [u] ulceration, [f] fistula, [a] abscess). Thus, the pufa index complements the dmft index by displaying the severity of dental decay and quantifying odontogenic infections of the pulp and surrounding tissues due to untreated caries.

Recently, the pufa index was used particularly in low- and middle-income countries to display the severity of oral health neglect (Table 1). The pufa prevalence varies from 24 % in Brazilian 6- to 7-year-olds [22] up to 85 % in 6-year-old children from the Philippines [21], indicating a huge dental need. Although Germany is a high-income country, the treatment of dental decay in the primary dentition of pre-school children is insufficient. The last representative study amongst 6- to 7-year-old German children revealed that almost half of all carious primary teeth (47.4 %) are untreated [7]. This situation has been nearly unchanged for more than 10 years [23]. Until now, the consequences of untreated dental caries—odontogenic infections—had not been quantified in German children with the pufa index. Therefore, the aim of this cross-sectional study was to assess the prevalence and experience of caries and odontogenic infections in primary teeth of 5- and 8-year-old German children from the Westphalian Ennepe-Ruhr District (EN District). The null hypotheses tested were as follows: 1) there is no difference in the prevalence and experience of odontogenic infections between 5- and 8-year-old German children; 2) untreated dental caries does not correlate with odontogenic infections; 3) odontogenic infections in 8-year-olds do not correlate with caries experience in permanent teeth.

**Table 1:** Pufa prevalence and mean pufa index in the primary dentition of children—overview from the literature

Authors	Year of publication/ investigation	Population N/ country	Age of Population	dmft prevalence (%)	dmft (mean ± SD)	pufa prevalence (%)	pufa (mean ± SD)
Mehta A., Bhalla S.	2014/2014	N = 603/ Indian	5–6 years	69.5	not reported	38.6	0.9 ± 1.93 pufa
							0.84 ± 1.5 p
							0.001 ± 0.05 u
							0.01 ± 0.08 f
							0.5 ± 0.3 a
Baginska J., et al.	2013/2011	N = 215/ Poland	5 years	85.9	5.56 ± 4.45 dmft	43.0	2.20 ± 3.43 pufa
					4.90 ± 4.26 dt		2.13 ± 3.35 p
					0.19 ± 0.83 mt		0.00 u
					0.46 ± 1.16 ft		0.07 ± 0.33 f
							0.00 a
			7 years	94.8	6.69 ± 3.14 dmft	72.0	2.44 ± 2.22 pufa
					5.22 ± 3.25 dt		2.31 ± 2.14 p
					0.86 ± 1.33 mt		0.01 ± 0.09 u
					0.61 ± 1.24 ft		0.12 ± 0.35 f
							0.00 a
Thekiso, M., et al.	2012/2010	N = 800/South Africa	4–5 years	49	:2.4 dmft	33.0	2.9 ± 2.4 pufa
					2.0 dt		1.8 ± 1.9 p
					0.2 mt		0.7 ± 0.4 u
					0.2 ft		0.0 f
							0.4 ± 0.1 a
			6–8 years	46	2.4 dmft	41.0	3.4 ± 3.9 pufa
					2.0 dt		2.9 ± 2.6 p
					0.3 mt		0.3 ± 0.6 u
					0.1 ft		0.1 ± 0.4 f
							0.1 ± 0.3 a
Figueiredo M.J., et al.	2011/2009	N = 835/ Brazil	6–7 years	not reported	not reported	23.7	0.4 ± 0.9 pufa
							0.3 ± 0.7 p
							0.001 ± 0.03 u
							0.08 ± 0.3 f
							0.01 ± 0.1 a
Monse B., et al.	2012/2006	N = 2030/ Philippines	6 years	96.8	8.4 ± 4.2 dmft	85.0	3.4 ± 2.6 pufa
					8.0 dt		2.9 ± 2.4 p
					0.4 mt		0.3 ± 1.0 u
					0.0 ft		0.1 ± 0.4 f
							0.1 ± 0.3 a

## Methods

### Study population

Data from oral examinations provided by public health service between January and December 2011 to 1.104 children aged five (*n* = 496) and eight (*n* = 608) years living in the EN District in Germany were included in this cross-sectional epidemiological study. By the law, free dental screenings to all children and adolescents attending preschools and schools are annually offered by the Department of Social Services and Health, Health Services for Children and Adolescents of the EN District. The age group of 5-year-olds was chosen because of a good comparability to international studies regarding the pufa index (Table 1) and the possibility to evaluate ECC. The attendance of pre-schools for 5-year-olds is highly recommended by the German Department of Education. Therefore, the large majority of 5-year-olds is visiting pre-schools, enabling the examination of a representative sample of children. Furthermore, 5-year-olds display the highest stage of the complete developed primary dentition before the first permanent teeth are erupting.

At the age of eight, children reveal the end of the second phase of the mixed dentition—before exfoliation of the primary molars—and the first permanent molars are commonly erupted and exposed to the oral environment since 2 years. Therefore, examination of 8-year-olds enables to show the influence of caries experience in the primary dentition on dental health of the permanent dentition; particularly the first permanent molar.

The EN District is located in the centre of the federal state North Rhine-Westphalia (NRW) in Western Germany. NRW experienced high industrialisation and urbanisation in the early 20th century, becoming the largest conurbation and centre of coal industry in Europe. Because of the coal-crisis in the years following 1960, the socio-economic status declined. In 2011 NRW had an at-risk-of-poverty rate of 14.6 % which is comparable to Germany (15 %) [24]. The report of poverty for the EN District in 2010 states that this district is representative for NRW showing a slightly higher at-risk-of-poverty rate (16 %) [25].

The sample size was estimated to the number of children necessary to obtain statistical significance with 80 % power and an interval of 5. Targeting the estimated sample size and following the regional socio-demographic pattern, 21.0 % (34 out of 162) pre-schools and 38.2 % (21 out of 55), primary schools were selected from the different areas. Selection criteria of the schools/pre-schools included area (urban/rural and industrialized/middle-class), size (small and large), ownership (public/private) and socio-economic status targeting a proportional distribution.

The exclusion criteria for the cross-sectional study were: 1) absenteeism from school/pre-school 2) child has a special health care need and 3) refusal of the child to be examined in the pre-school setting. Eighty per cent (495 out of 620) of all 5-year-olds attending the selected pre-schools and 88.9 % (608 out of 684) of all 8-year-olds attending the selected primary schools could be included in this study.

### Examiner calibration

Prior to the survey, the examiner (I. G.) received 1-day theoretical and clinical calibration training for using the dmft and pufa indices. An experienced dentist and epidemiologist (R. H.-W.) conducted the training. Ten children were examined in a pre-school and a primary school not included in this survey, but under the same field conditions as in the main study. The intra- and inter-examiner-reproducibility was assessed by the kappa (κ) statistics. The κ values for inter-examiner-reproducibility ranged from 0.90 (I. G.) to 0.92 (R. H-W.) for the pufa index, demonstrating excellent agreement, and values for inter-examiner reproducibility for the dmft was in the same range (0.88 I. G/0.93 R. H-W.). Intra-examiner reproducibility ranged from 0.89 to 1.00 for the examiner (I. G.) for both indices. Within the main study, every 20th child was repeatedly examined. The intra-examiner reproducibility for both indices ranged between 0.91 and 1.00.

### Oral examinations

The examinations were performed by one calibrated dentist in classrooms in each pre-school or primary school (I. G.). The caries status of the children was assessed according to WHO criteria [20] using an intra-oral mouth mirror, a CPI ball-end probe, and a halogen examination light (Mach 113, Dr. Mach GmbH & Co. KG, Ebersberg, Germany) after tooth-brushing supervised by the dental nurse or teacher. Cotton rolls were used for moisture control. Caries was assessed using the dmft index and the dmft/DMFT in 8-year olds.

The clinical consequences of untreated caries were recorded by using the pufa index [21]. The pufa index per child represents the number of teeth meeting the following diagnostic criteria: Decayed teeth with visible pulpal involvement (p) was measured when the open pulp chamber was visible or the clinical crown was destroyed and only root fragments were left. Ulceration (u) of the soft tissue surrounding the tooth was scored when caused by dislocated tooth fragments. Fistula (f) was diagnosed when pus-releasing sinus tract was related to the tooth with pulpal involvement. Abscess (a) was scored when a pus-containing swelling was related to the tooth. The diagnosis of the pufa index was performed visually, without the use of a dental probe.

### Statistical analysis

Data collection was performed with excel spreadsheets (Excel 2011 Microsoft Cooperation, Redmond, WA, USA) and the statistical analysis of the oral health data was carried out using SPSS 21.0. (IBM Corp, Armonk, NY, USA), R 3.1.1 (R Core Team, 2014 [26]). Caries experience was calculated as mean dmft and the Significant Caries Index (SiC index) as the mean dmft of one third of the population with the highest caries scores [27]. ECC in 5-year-olds was assessed according to the definition of Wyne differentiating between mild (type I), moderate (type II) and severe caries pattern (type III) [18]. The Lorenz curve was used to display the polarisation of dental caries (cumulative disease), and the extent of inequality was measured by the Gini coefficient (G) with finite population correction. The range of the Gini coefficient is 0 ≤ G ≤ 1, with value 0 indicating equality and value 1 expressing maximal inequality. The care index was calculated as [ft/dmft] × 100. The severity of untreated dental caries was recorded by the pufa index. The ‘untreated caries-pufa ratio’ was calculated as [pufa/dt] × 100 and describes the percentage of untreated carious teeth that developed an oral infection. The correlation between dmft and pufa scores was computed by the Spearman’s rank correlation coefficient (ρ). Categorical data were compared between groups using the chi-square test and continuous data were analysed by t-test. A binary logistic regression model was fitted to determine the influence of dmft and pufa on the risk of developing dental caries in the permanent dentition. Statistical significance level was set at *p* ≤ 0.05.

### Ethical considerations

This study was performed in full accordance with ethical principles and approved by the ethics committee of the Jena University Hospital (registration number 3660-D1/13).

## Results

### 5-year-old children

**Fig. 1:**
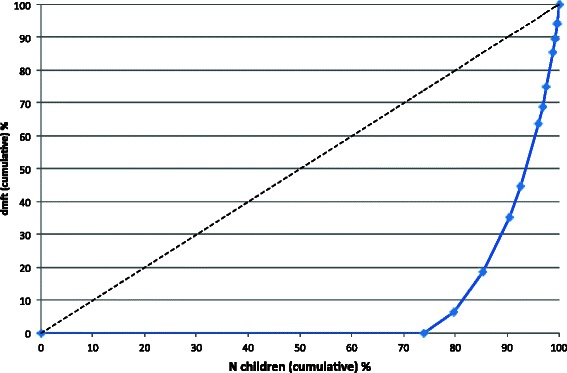
Lorenz curve for the dmft distribution of 5-year-old German children

**Fig. 2:**
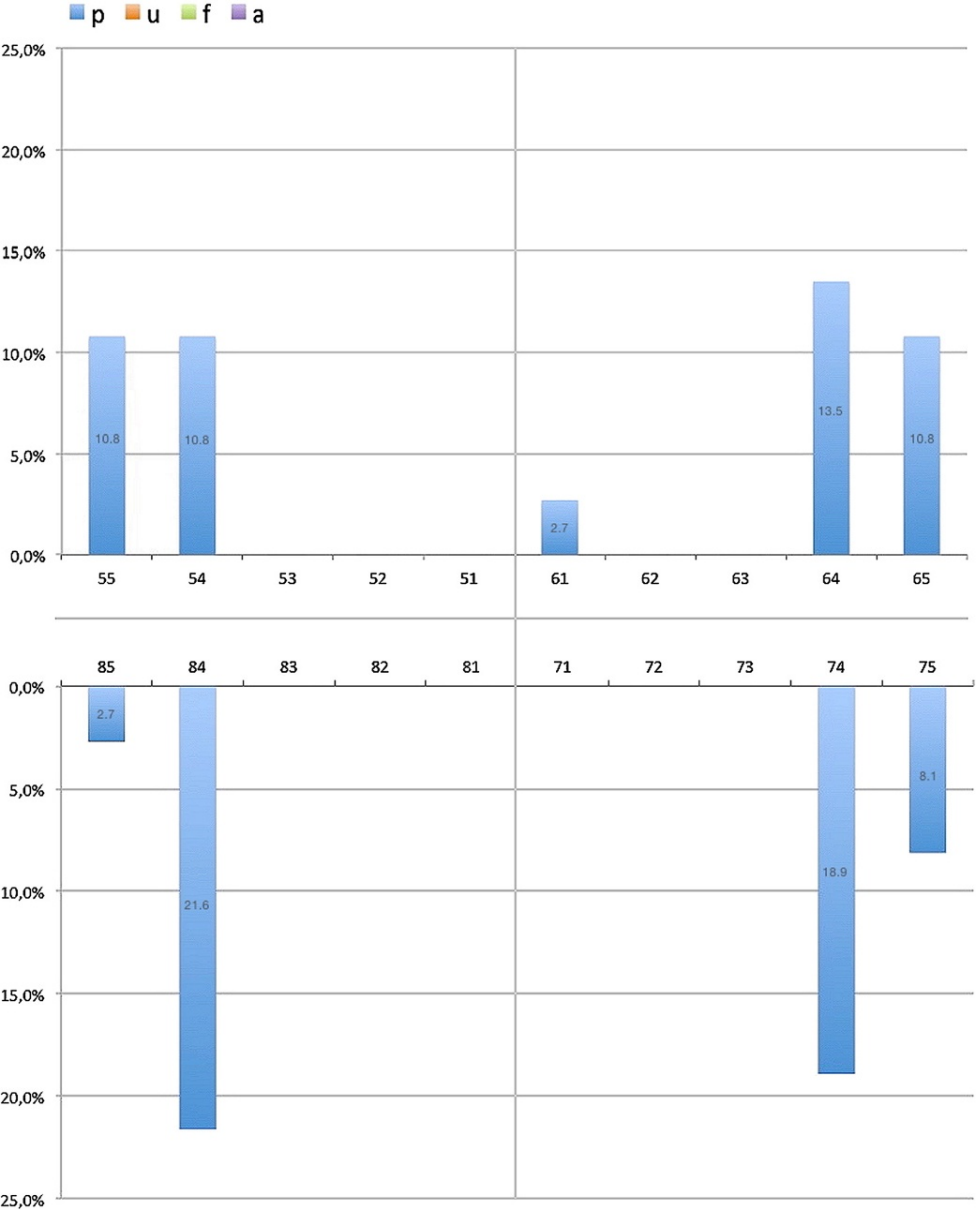
Tooth related distribution of the single pufa components in German 5-year-olds

Four hundred and six 5-year-olds (249 boys) with an average of 19.4 primary and 0.7 permanent teeth were included in the analysis. Caries prevalence was 26.2 % and caries experience was 0.9 ± 2.0 dmft (Table 2). The SiC index amounted to 2.8 dmft. ECC was distributed to 22 % on ECC type I, 4 % on type II and 0.2 % on type III. Caries polarisation is displayed by the Lorenz curve in Fig. 1 and confirmed by the high Gini coefficient (G = 0.84). The care index was 29.7 %, indicating that less than one-third of the dental decay was treated. The prevalence of odontogenic infections was 4.4 % and exclusively concentrated on pulpal involvement (p) with a mean pufa of 0.1 ± 0.5 (Table 2). Boys had a higher pufa (0.1 ± 0.7) than girls did (0.0 ± 0.2, *p* = 0.035) and also had a higher untreated caries-pufa-ratio (boys = 20.4 %, girls = 6.1 %, *p* = 0.030). First primary molars were affected most frequently by odontogenic infections (Fig. 2). Nearly all dental decay (93.6 %) and odontogenic infections (89.2 %) were concentrated in 20 % of the children, showing a significant correlation between high dmft and pufa scores (*ρ* = 0.399, *p* < 0.001).

**Table 2:** Prevalence and experience of dental caries and odontogenic infections in the primary dentition

		5-year-olds				8-year-olds		
	Total	Boys	,.Girls	*p*-value	Total	Boys	Girls	*p*-value
Number	496	249	247		608	298	310	
Caries prevalence (%) [95 % CI]	26.2 [22.5–30.3]	26.1 [21–31.9]	26.3 [21.2–32.1]	1.000	48.8 [44.9–52.8]	51.5 [45.7–57.0]	46.1 [40.7–51.7]	0.194
dmft x ± SD	0.9 ± 2.0	1.0 ± 2.2	0.8 ± 1.8	0.255	2.1 ± 2.8	2.3 ± 2.9	1.8 ± 2.6	0.032*
dt x ± SD	0.5 ± 1.4	0.6 ± 1.6	0.5 ± 1.2	0.316	0.9 ± 1.7	1.1 ± 1.8	0.8 ± 1.6	0.074
mt x ± SD	0.1 ± 1.8	0.2 ± 1.1	0.0 ± 0.3	0.052	0.3 ± 1.0	0.4 ± 1.1	0.3 ± 1.0	0.189
ft x ± SD	0.3 ± 1.0	0.2 ± 0.8	0.3 ± 1.1	0.464	0.8 ± 1.6	0.9 ± 1.7	0.8 ± 1.5	0.348
SiC x ± SD	2.8 ± 2.7	3.1 ± 3.0	2.5 ± 2.3	0.256	5.6 ± 1.9	6.1 ± 1.9	5.0 ± 2.0	0.056
Care index (%) [95 % CI]	29.7 [25.7–34]	23.6 [18.8–29.2]	37.3 [30.9–44.2]	0.338	39.3 [36.6–42]	37.9 [34.3–41.6]	41.1 [37.1–45.2]	0.302
pufa prevalence (%) [95 % CI]	4.4 [2.9–6.6]	6.0 [3.7–9.7]	2.8 [1.4–5.7]	0.125	16.6 [13.9–19.8]	21.2 [16.9–26.1]	11.9 [8.8–16.0]	0.002^+^
pufa x ± SD	0.1 ± 0.5	0.1 ± 0.7	0.0 ± 0.2	0.035*	0.3 ± 0.9	0.4 ± 1.0	0.2 ± 0.8	0.008*
p x ± SD	0.1 ± 0.5	0.1 ± 0.7	0.0 ± 0.2	0.035*	0.3 ± 0.9	0.4 ± 1.0	0.2 ± 0.8	0.016*
u x ± SD	0.0 ± 0.0	0.0 ± 0.0	0.0 ± 0.0	-	0.0 ± 0.1	0.0 ± 0.2	0.0 ± 0.0	0.021*
f x ± SD	0.0 ± 0.0	0.0 ± 0.0	0.0 ± 0.0	-	0.0 ± 0.0	0.0 ± 0.0	0.0 ± 0.0	-
a x ± SD	0.0 ± 0.0	0.0 ± 0.0	0.0 ± 0.0	-	0.0 ± 0.1	0.0 ± 0.0	0.0 ± 0.1	0.158
Untreated caries pufa ratio (%) [95 % CI]	14.2 [10.5–18.9]	20.4 [14.7–27.6]	6.1 [3.0–12.1]	0.030*	34.2 [30.4–34.2]	39.3 [34.0–44.8]	27.6 [22.4–33.4]	0.003*

*p*-value statistically significant (^+^chi-square test, *t-test)

### 8-year-old children

**Fig. 3:**
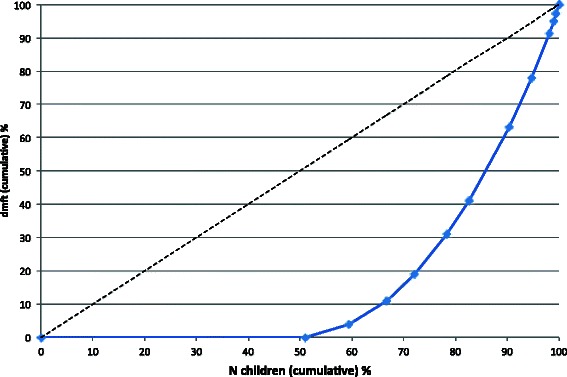
Lorenz curve for the dmft distribution of 8-year-old German children

Six hundred eight 8-year-olds (298 boys) with 11.3 permanent and 11.7 primary teeth were examined. Caries prevalence was 48.8 % in the primary (Table 2) and 3.9 % in the permanent teeth. The caries experience of the primary teeth was 0.9 ± 2.0 dmft and of the permanent teeth 0.1 ± 0.4 DMFT. The SiC index amounted to 5.6 ± 1.9 dmft and 0.2 ± 0.7 DMFT, respectively. Caries polarisation shown by the Lorenz curve revealed that 66 % of the total caries experience was concentrated on 20 % of the children (Fig. 3). The Gini coefficient was 0.67, showing a lower concentration than in the population of the 5-year-olds. The care index of the primary teeth was 39.3 % with no significant difference between boys and girls.

**Fig. 4:**
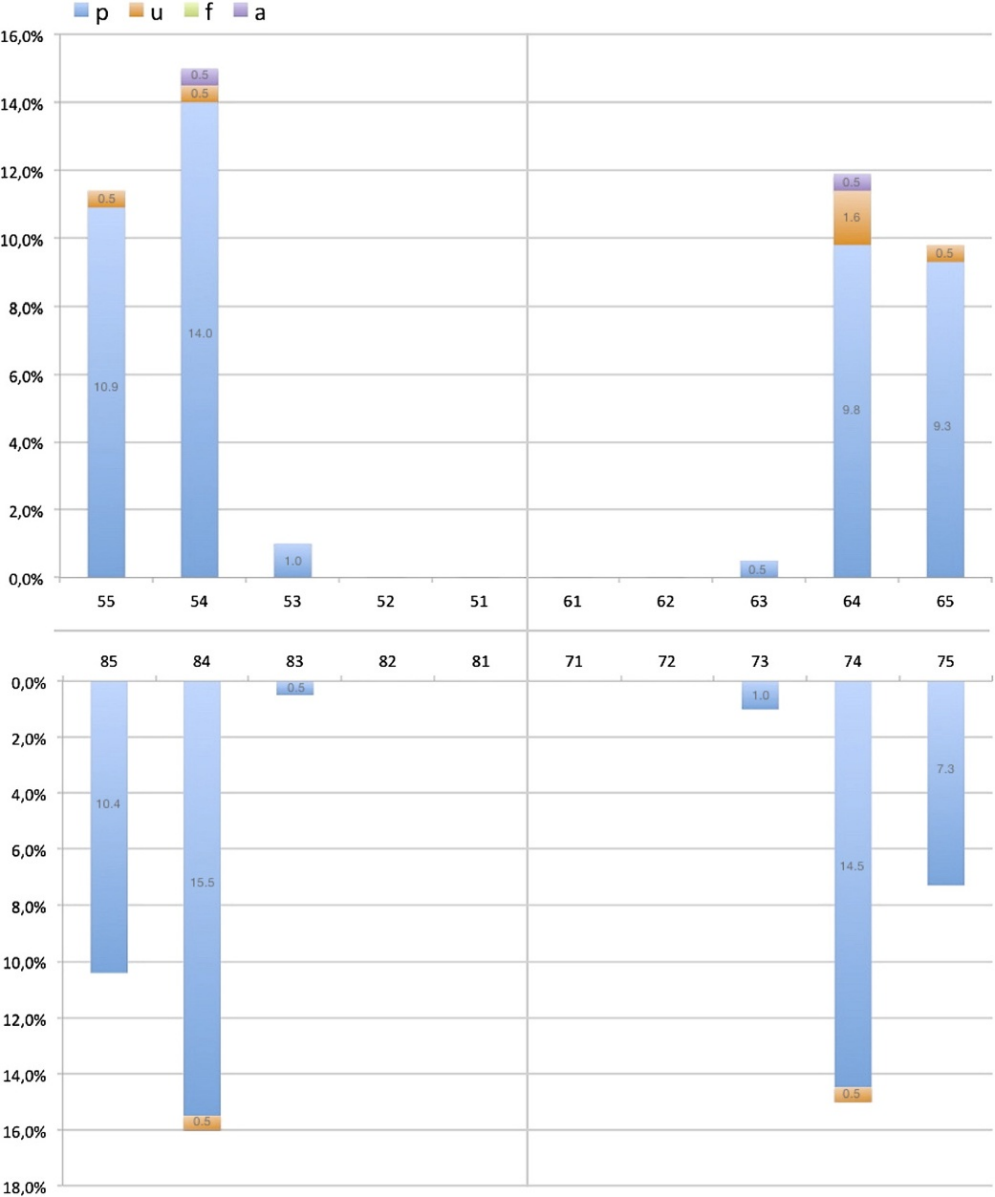
Tooth related distribution of the single pufa components in German 8-year-olds

Pufa prevalence amounted to 16.6 % and the mean pufa was 0.1 ± 0.5 (Table 2). Pulpal involvement (p) was scored most frequently (94.8 %). The untreated caries-pufa-ratio indicates that 34.2 % of the d-component had progressed mainly to the pulp, indicating a significant correlation between dmft and pufa scores (*ρ* = 0.499, *p* < 0.001). The prevalence of caries experience and odontogenic infections was significantly higher in boys. Almost 40 % of untreated caries in boys revealed a pufa index >0. Figure 4 presents the tooth related distribution of single pufa components in 8-year-olds. Virtually all pufa scores (96.9 %) were concentrated on primary molars with pulp involvement and the ulceration of soft tissues. The first primary molars were the most affected teeth. Twenty-four (3.9 %) of all examined 8-year-olds revealed caries experience in permanent teeth (DMFT > 0). Of these children, 41.7 % also showed a pufa score in the primary dentition (Table 3). Caries experience in the permanent dentition was significantly determined by dmft index and the dt and mt component as well as by pufa index and the p component (t-test). For each unit increase in dmft and pufa the risk of caries experience in the permanent dentition increased by 33.9 % (OR 1.339, *p* = 0.00) and 34.9 % (OR 1.349, *p* = 0.03) (Table 4).

**Table 3:** Dental caries and odontogenic infections in the primary dentition of 8-year-olds with and without caries experience in the permanent dentition

8-year-olds				
	Total	DMFT = 0	DMFT > 0	*p*-value
Number	608	584	24	
Caries prevalence (%) [95 % CI]	48.8 [44.9–52.8]	47.3 [43.2–51.3]	87.5 [69.0–95.7]	<0.001^+^
dmft x ± SD	2.1 ± 2.8	1.9 ± 2.7	5.5 ± 3.1	<0.001*
dt x ± SD	0.9 ± 1.7	0.8 ± 1.6	2.9 ± 3.3	0.006*
mt x ± SD	0.3 ± 1.0	0.3 ± 1.0	1.3 ± 1.4	0.003*
ft x ± SD	0.8 ± 1.6	0.8 ± 1.6	1.4 ± 2.0	0.172
pufa prevalence (%) [95 % CI]	16.6 [13.9–19.8]	15.6 [12.9–18.7]	41.7 [24.5–61.2]	0.003^+^
pufa x ± SD	0.3 ± 0.9	0.3 ± 0.8	1.5 ± 2.4	0.015*
p x ± SD	0.3 ± 0.9	0.3 ± 0.7	1.5 ± 2.4	0.021*
u x ± SD	0.0 ± 0.1	0.0 ± 0.1	0.1 ± 0.4	0.390
f x ± SD	0.0 ± 0.0	0.0 ± 0.0	0.0 ± 0.0	-
a x ± SD	0.0 ± 0.1	0.0 ± 0.1	0.0 ± 0.0	0.774

*p*-value statistically significant (*t-test, ^+^Fisher’s exact test)

**Table 4:** Dental caries and odontogenic infections in the primary dentition of 8-year-olds as risk factors for dental caries in permanent teeth

	Regression Coefficient	*p*-value	OR	95 % CI
dmft	0.292	<0.001*	1.339	1.151–1.557
pufa	0.299	0.030*	1.349	1.025–1.775

*p*-value statistically significant (*binary logistic regression analysis)

## Discussion

In recent years, epidemiological caries research in high-income countries like Germany focussed on the development of more sensitive diagnostic methods to enable the assessment of initial caries lesions like the International Caries Detection and Assessment System (ICDAS II) [28, 29]. This trend results from the decline of cavitated caries lesions and the development of non-invasive and preventive interventions requiring a distinction between different stages of initial caries lesions. In contrast, the remaining decay is concentrated in a small group of children with high caries levels and a huge need for treatment [27]. Epidemiological studies have indicated that socio-economic conditions are important risk factors for caries during childhood [30, 31]. Thus, high caries prevalence and experience in low-income countries [32] and in socio-economically disadvantaged groups [33, 34] have been documented. That polarisation phenomenon is also present in German children [35–37]. Our findings display the inequality of caries distribution by the Lorenz curve (Fig. 1), confirmed by the Gini coefficient. In 5-year-olds, 90 % of the whole caries burden was concentrated in 20 % of the children, showing a strong polarisation of ECC. Unfortunately, no data on the children’s ethnical or socio-economic background were collected in the examination, which is a limitation of this survey.

Untreated caries may affect seriously the quality of children’s life because of pain and discomfort, which could lead to acute and chronic infections, oral mucosal conditions and altered eating and sleeping habits [38, 39]. Furthermore, untreated caries in primary teeth can have a lasting detrimental impact on the permanent dentition by causing high caries risk [40] or developmental defects of the permanent successor tooth [41]. This was demonstrated in the present study population by the fact that 41.7 % of 8-year-olds with dental decay in the permanent dentition (DMFT > 0) also presented pufa scores in the primary dentition (pufa > 0). With this fact the third null hypothesis that there is no correlation of odontogenic infections and caries in the permanent dentition of 8-year-olds was rejected (Table 3). Thus, children with pufa scores should be characterized to be at high caries risk for early caries onset in permanent teeth. Presenting only dmft data to decision makers leaves them unaware of the severity and associated consequences of untreated caries on general and dental health [10, 42]. Therefore, for the first time, our study gathered data on odontogenic infections as consequences of untreated dental caries in 5- and 8-year-old German children by using the pufa index.

About one-third to one-half of Westphalian 5- to 8-year-olds suffered from caries in primary teeth, which is in the same range (47.3 %) estimated for 6- to 7-year-olds in the last representative epidemiological German study in 2009 [7]. About one third of all decay was restored (5y: 29.7 %; 8y: 39.3 %), leaving the teeth to development of pulpal involvement and odontogenic infections. This reflects the fact, that many German dentists perceive dental treatment of children as stressful [43]. Commonly, dental school graduates are insufficiently qualified because of the limited university education in paediatric dentistry. Furthermore, for most dentists, it is not attractive to attend a postgraduate paediatric curriculum, due to inadequate reimbursement for restorative treatment in small and pre-school children with limited or lacking capability to cooperate [43]. But the risk of young children experiencing pain and sepsis increases with higher caries experience [4]. Therefore, children at high caries risk would benefit most from early dental care. On average, every twentieth 5-year-old child (4.4 %) and every sixth 8-year-old child (16.6 %) had odontogenic infections. Thus, the first the null hypothesis that there is no difference in the prevalence and experience of odontogenic infections between 5- and 8-year-old German children was rejected. In 5-year-olds, nearly all odontogenic infections (89 %) were concentrated in 20 % of the children displaying the highest dmft scores. This is emphasised by the correlation of untreated dental caries and odontogenic infections (*ρ* = 0.399, *p* < 0.001). Hence, the second null hypothesis that untreated dental caries does not correlate with odontogenic infections was rejected.

Taking into account the different socio-economic background of the population examined, comparisons with other countries may be limited. The pufa prevalence of our population was considerably low (5y: 4.4 %, 8y: 16.6 %) compared to Filipino 6-year-olds (85 %) [21], Brazilian 6- to 7-year-olds (23.7 %) [22], South African 4- to 5-year-olds (33 %) and 6- to 8-year-olds (41 %) [44], Polish 5-year- (43 %) and 7-year-olds (72 %) [45], and Indian 5- to 6-year-olds (38.6 %) [46]. Solely, the pufa prevalence in 8-year-old German boys (21.2 %) was in the same range as reported for Brazilian children [22] since 40 % of the caries lesions had progressed to the pulp. Knowing the impact of severe consequences of untreated dental caries on children’s general health, these findings should lead to the development of programs for German children at high caries risk.

Consistent with the studies mentioned, pulp involvement (p) was the diagnosis most frequently recorded, followed by ulceration (u) especially in 8-year-olds. This is in contrast to the findings of Figueiredo et al. [22] and Baginska et al. [45], revealing different patterns of odontogenic infections in different countries. The fact that primary molars were the teeth most affected by pulp involvement is consistent with their high caries susceptibility [45–47]. Possible causes for children showing more odontogenic infections in first primary molars are their earlier eruption compared to the seconds which leads to a longer oral cariogenic exposure and the potential of lesions development between eruption and examination time. Furthermore the faster lesion progression from enamel surface to the dental pulp due to the lower enamel-dentin-thickness is related to larger pulp chambers compared to second primary and permanent molars [48, 49]. Additionally, the early age of the child at eruption of first primary molars as well as their posterior position at the dental arches may contribute to more difficult and less efficient tooth brushing by parents or care givers. However, there is no consistent evidence that the first primary molars are more often carious affected [46, 50] than the second primary molars [51-54].

Frigueiro et al. [22] and Murthy et al. [55] suggest that the codes ‘f’ and ‘a’ of the pufa index could be grouped together since they refer to the same inflammatory process of the jaw bone and are only different stages of inflammation. Furthermore, the necessity to score these codes separately was questioned, as the treatment requested will be the same: endodontic treatment or extraction [56, 57]. In this context, it should be considered that the pufa index was not designed to serve as a treatment need index, but rather as an index to quantify the severity of untreated dental caries and to assess the presence of odontogenic infections [58].

The use of only the dmft/DMFT index may be misleading the interpretation of caries epidemiological data. That was shown by the national oral health survey of the Philippines [21] reporting 2.9 DMFT in 12-year-olds, which fulfils the WHO/ FDI goal of 3 DMFT for this age group in 2000 [59]. However, in reality, 41 % of the decay component had progressed to odontogenic infections assessed by the PUFA index, indicating the huge severity of untreated tooth decay. The dmft/DMFT index fails to provide information on the clinical consequences of untreated dental caries, which may be more serious than the caries lesions themselves. The more meticulous caries classification system, ICDAS II enables the recording of different caries progression stages from sound to extensive decay compared to the dmft/DMFT [26]. However, scoring of odontogenic infections (pufa/PUFA index) is only optionally recommended. Until now, German public oral health services prefer using dmft/DMFT index, considering ICDAS II too complicated and time consuming. The new caries assessment spectrum and treatment (CAST)-index was developed combining elements of the ICDAS II and the pufa/PUFA index with the m- and f-components of the dmft/DMFT index [60]. It covers the total spectrum of carious lesion progression, including the advanced stages of carious lesion progression in the pulpal and tooth surrounding tissue. De Souza et al. compared the assessment of dental caries using the CAST instrument and the DMFT index showing no difference between the recorded caries prevalence, caries experience and time spent for examination [61]. Still there is a need to validate the CAST index more closely before trying to replace any other index.

Dental caries is a multifactorial chronic disease with the interplay of individual, cultural, social and socio-economic risk factors. The lack of data regarding these factors is a limitation of the present cross-sectional study. Nevertheless, using the pufa index provides a more comprehensive view on caries pattern in primary teeth of German children. However, there is a lack of other studies performed in high-income countries to compare the findings.

## Conclusion

This is the first German survey showing prevalence and experience of odontogenic infections as consequences of severe untreated dental caries in primary teeth among German 5- and 8-year-olds by using the pufa index. Prevalence and experience of odontogenic infections and the untreated caries-pufa ratio were increasing from the younger to the elder children. Pufa scores in primary teeth predict a higher caries risk in permanent teeth. The pufa index highlights relevant information by assessing the severity of untreated dental caries for dentists and decision makers to develop effective oral health care programs for children at high caries risk.

## Abbreviations

AAPD: American Academy of Pediatric Dentistry
CAST: Caries assessment spectrum and treatment
CPI: Community Periodontal Index
DMFT: Decayed, missing, filled tooth
ECC: Early childhood caries
EN: Ennepe-Ruhr
FDI: World dental federation
ICDAS II: International Caries Detection and Assessment System
NRW: North Rhine-Westfalia
PUFA: Pulpal involvement, ulceration, fistula, abscess
SiC: Significant caries
WHO: World Health Organisation
